# Correction: The effectiveness of problem based learning in improving critical thinking, problem-solving and self-directed learning in first-year medical students: A meta-analysis

**DOI:** 10.1371/journal.pone.0303724

**Published:** 2024-05-09

**Authors:** Ida Bagus Amertha Putra Manuaba, Yi -No, Chien-Chih Wu

There is an error in affiliation 1 of the first author. The correct affiliation 1 of Ida Bagus Amertha Putra Manuaba is: International Ph.D. Program in Medicine, College of Medicine, Taipei Medical University.
