# Supplementary material for: The effectiveness of problem based learning in improving critical thinking, problem-solving and self-directed learning in first-year medical students: A meta-analysis
Source: PLoS One. 2022 Nov 22;17(11):e0277339. doi: 10.1371/journal.pone.0277339 (PMC9681085; doi:10.1371/journal.pone.0277339)
Supplement: Attachment — Submitted filename: Response to reviewers(1).docx [file pone.0277339.s002.docx]

**RESPONSE TO REVIEWERS**

**Dear reviewers**

**Thank you very much for your reviews and critics. We have made several changes in our manuscript according to your review points. We hope that we have met your standards and requirements. If there is anything that we need to change, please do not hesitate to inform us, and we value your critics to improve our paper.**

**Below is the response that we made, and the changes have been highlighted in our paper.**

**Reviewer #1**

1. **Title**

**The study titles as 'The effectiveness of problem-based learning in the education of first-year medical students in multidisciplinary aspects: a meta-Analysis. However, the objective is to synthesize the effectiveness of PBL in improving knowledge/critical thinking, problem-solving and self-directed learning in first-year medical students. The title didn't reflect the study exactly. Hence, a revision is suggested. Also the term multidisciplinary approach may lead mislead the readers**

The word “multidisciplinary” has been removed from the title because there was no discussion regarding the differences in the effectiveness of PBL in each medical study program

Title revision:

“The effectiveness of Problem Based Learning in improving critical thinking, problem-solving and self-directed learning in first-year medical students: a meta-Analysis”

2. **Abstract**

**At this point of time, Problem based learning (PBL) is not an innovative method as it is used widely used as a constructivist pedagogic philosophy and an instructional format for promoting contextual, co-operative and self-directed learning. Moreover, PBL is used not only for medical education. Please revise the statement. The authors are encouraged to discuss the challenges faced by the first year medical students in adapting PBL to justify the study objective. The reasons such as current studies focused on students in advanced years and lack of such studies in the first-year students should not be the only reason for this study.**

The author has revised the statement that “PBL is an innovative method”. The author focuses on assessing the advantages of PBL compared to conventional learning methods for first-year medical students due to the unclear effect of this learning method in this population.

3. **Introduction**

- **The authors' statement on the effectiveness of PBL "Although PBL-used widely, still there is no substantial evidence to support the claim that PBL is proven to be more effective than conventional lecture methods" merely highlighting the comparativeness between PBL and Lecture. In fact, there are reports on the effectiveness of PBL, which improves student engagement by enabling knowledge and information sharing and discussion. The delivery of curriculum in medical education has a variety of methods to teach. It would be appropriate if authors focus more on how PBL is compared with other forms of teaching as well.**

The author has added a sentence that focuses more on the effectiveness of PBL compared to other forms of teaching methods (especially conventional learning methods, which are used as comparisons in this study).

- **The last paragraph of introduction, explained the results of 2 studies [5,6] and mentioned that there has been no meta-analysis that discusses the effectiveness of PBL in first-year health students in multidisciplinary aspects. What does it mean by health students? 🡪**

The health student that the author means is a medical student. The medical student is a student enrolled at a medical school, including a doctor, dentist, nurse, physiotherapist, or midwife. If there is any difference in our perspective, please let us know so we can revised the terms in this manuscript.

- **This paragraph need revision. Highlighting the connection between the research gap and the study objective clearly. The purpose of this meta-analysis is to synthesize the evidence on the effectiveness of PBL. A revision is required in that statement mentioned about the purpose as the meta-analysis is to examine an effect within a collection of studies.**

The author has revised the study objectives according to the research gap

4. **Methodology**

- **Eligibility criteria and data extraction is not clear.**
- We have adjusted the eligibility criteria and data extraction to make it clearer. Therefore, the eligibility criteria and data extraction were as follows:
- **The inclusion criteria for this study included**: (1) research subjects were medical students at the first year (first or second semester), (2) study that evaluated the knowledge/critical thinking, problem-solving and self-directed learning of the student, (3) studies that providing sufficient data for calculation of mean difference and 95%CI, p-value, and study heterogeneity. Meanwhile, the **exclusion criteria** were as follows: (1) studies with insufficient data, (2) samples less than 50, (3) intervention less than 1 year, (4) review, letter to the editor, and comments articles. **Data extraction** was conducted by two authors (I.BA.P.M, Y.N). Both authors independently screened the collected article's title, abstract, and full text. Two reviewers extracted the data was then extracted to Google Spreadsheet by two reviewers (I.BA.P.M, Y.N). Information was derived from each article included in this study as follows: (1) first author's name and year of release, (2) age of the participant, (3) interventional and control method, (4) sample cases and control sizes, (5) country of study, (6) study program, (7) duration of PBL intervention, (8) score of PBL and control method. Two independent authors carried out data extraction to prevent human mistakes. If there is a disagreement, a discussion will be held to discuss the solution.
- **Under quality assessment, it is mentioned If the conflict has not been settled, a Senior Researcher consultation has been carried out. Please provide the detail of the reviewer.**
- We have clarified the roles of 2 authors who act as reviewers in assessing the quality of each article screened in this study. The quality assessment section that we have revised is as follows:
- The quality of the studies was assessed by two independent authors (I.BA.P.M, Y.N) to ensure each sample's validity and prevent the possible exaggeration of each study. The authors use major and minor criteria in assessing the risk of bias for quality assessment. There were 4 major and 4 minor criteria. The authors assigned 2 points each to the major criteria and 1 point each to the minor criteria, so the total score could be 12 points. If the article got 9-12 points, then it assigned as “low-risk bias”, if the article got 6-8 points, then it assigned as medium risk bias”, and if the article got < 5 points, then it assigned as “high risk of bias”. When there was a disagreement between two separate authors, a discussion was held. If the conflict has not been settled, the two authors discuss it with the third author (C.C.W).
- **Reference 10 is not a RCT- it is a Quasi-experimental research, which specifically lacks the element of random assignment to treatment or control. Reference 11 also a randomised cross-matched study. Please review accordingly.**
- Reference no 10 (Sangestani et al) 🡪 now become reference no 13 🡪 this study type has been adjusted to a randomized quasi-experimental study. Based on that article, the design was quasi-experimental, but randomization was carried out to determine the treatment group (PBL group)
- Reference no 11 (Hayashi et al) 🡪 now become reference no 14 🡪 this study type has been adjusted to a randomized cross-matched study.

5. **Discussion**

- The Discussion section is an important scientific component in a manuscript describing a meta-analysis, as the authors should discuss their current findings in the context of the available scientific literature and existing knowledge. The readers would expect possible reasons for the positive or negative results of the meta-analysis. As meta-analyses are usually synthesizing the existing evidence from multiple primary studies, authors can recommend key suggestions for conducting and/or reporting future primary studies.

Positive and negative results are discussed in the discussion. We highlight the revision with green color in the discussion. Suggestion for the further primary study was discussed in the last paragraph of the discussion (green highlight). Here we attached our suggestion below :

“……….In addition, primary research is still needed to evaluate the effectiveness of PBL. We suggest conducting the primary study about PBL by carrying it out at several different educational centers so that in addition to getting the results of the PBL learning evaluation, researchers can also compare between educational centers. Researchers can also evaluate aspects of the culture and technological progress of the local area that can be a supporting or hindering factor in the implementation of PBL. Because very rarely this aspect is raised in PBL research. Psychological aspects also need to be discussed, because medical students in the first year may still be adopted to the learning obtained so that it affects the PBL implementation process.”

6. **Conclusion**

The PBL method in the learning process is a breakthrough made to replace the conventional learning system, which has many weaknesses. The statement is not the conclusion from the study. The authors may discuss such points in the discussion. There is a lack of connection between the research question and conclusion. It is suggested to review the conclusion. We revised the conclusion according to the suggestion, here we attached the revision :

“In conclusion, according to our finding PBL learning method do not effective in improving critical thinking/knowledge, problem-solving and self-directed learning in first-year medical students. In addition, our meta-analysis had several limitations, such as only evaluating the learning outcomes in the first year and does not evaluate continuously in the following year. We could not equate the instruments used in PBL learning and did not evaluate specifically based on the study program. We could not assess the socio-demography that might contribute to their learning process, particularly in their social culture.”

- Also, there is no limitation is mentioned. Provide the list of limitations of this study.

The author has provided the limitation in the discussion. Meanwhile, according to this comment, we moved the study limitation to the conclusion

**Reviewer #2**

This meta-analysis paper aimed to synthesize the effectiveness of PBL in improving knowledge/critical thinking, problem-solving and self-directed learning in first-year medical students. However few points need to be addressed

1. Just two databases is often not acceptable for data search. Minimum three is required to make sure you haven't missed any publication. Author needs to use Scopus or any other database and do the search as well as update Prisma chart

The author has used more than two databases, namely Pubmed, ScienceDirect, Cochrane and Google Scholar.

2. Heterogeneity is very high in almost all forest plots. These needs to be explored and discussed more in detail

We have revised it and signed it with a green highlight

3. Authors need to critically evaluate the included articles in their discussion. The discussion is too superficial.

We have revised it and signed it with a green highlight

4. Few errors in grammar could be identified

The author has revised the grammar of this manuscript
