# Supplementary material for: The effectiveness of problem based learning in improving critical thinking, problem-solving and self-directed learning in first-year medical students: A meta-analysis
Source: PLoS One. 2022 Nov 22;17(11):e0277339. doi: 10.1371/journal.pone.0277339 (PMC9681085; doi:10.1371/journal.pone.0277339)
Supplement: Attachment — Submitted filename: Rebuttal Letter 2.docx [file pone.0277339.s003.docx]

REBUTTAL LETTER

Dear Editors and Reviewers,

Thank you for your corrections to our manuscript. We have made several revisions according to the reviewer’s comments and we hope that this manuscript is now met your standard. The revisions are as follows:

**Introduction**

1. The adaptation process for first-year medical students is an important problem because it is one of the factors that significantly affect educational outcomes. reference ?

Response: We have put the reference in the text.

2. A study on the adaptation process of first-year medical students involving 200 participants showed that 63% of students had problems adapting to the learning process. reference?

Response: We have put the reference in the text.

3. Problem-based learning (PBL) is an educational method that emphasizes active participation, problem-solving, and critical thinking skills compared to conventional medical education practices.[3] This statement is contradictory to the results. Ideally authors can consider to keep this point the later stage, mostly at the end of the paragraph rather than starting with this.

Response: We have revised this section and moved it to the end of paragraph.

**Discussion**

4. Various studies have been conducted regarding the effectiveness of PBL to be applied in
   274 the teaching and learning process. – References?

Response: We have put the references

5. Meanwhile, intervention duration might not be an absolute factor of PBL effectiveness because Tripathi [16] found significant results, and this study had the shortest intervention duration compared to other studies. This is statement is not clear.

Response: We have revised the statements

6. In this meta-analysis, we evaluated medical students in their first year. - Actually the authors evaluated the data only. Please revise

Response: We have revised the statement

7. First-year students often experience obstacles is adapting to lecture methods that are different from high school teaching methods. reference?

Response: We have put the reference

8. Several studies discuss the PBL effect on general learning outcomes and specific backgrounds. What are the studies ? references

Response: We have put the references

9. Under conclusion section, authors mentioned that "In conclusion, according to our findings",-doesn't sound logical. Pls consider to revise.

Response: We have revised the statement

Best Regards,

Chien-Chieh Wu
